# A Pedestrian Detection Network Model Based on Improved YOLOv5

**DOI:** 10.3390/e25020381

**Published:** 2023-02-19

**Authors:** Ming-Lun Li, Guo-Bing Sun, Jia-Xiang Yu

**Affiliations:** College of Electronics Engineering, Heilongjiang University, Harbin 150080, China

**Keywords:** pedestrian detection, lightweight model, global attention mechanism, Ghost modules, loss function

## Abstract

Advanced object detection methods always face high algorithmic complexity or low accuracy when used in pedestrian target detection for the autonomous driving system. This paper proposes a lightweight pedestrian detection approach called the YOLOv5s-$G^{2}$ network to address these issues. We apply Ghost and GhostC3 modules in the YOLOv5s-$G^{2}$ network to minimize computational cost during feature extraction while keeping the network’s capability of extracting features intact. The YOLOv5s-$G^{2}$ network improves feature extraction accuracy by incorporating the Global Attention Mechanism (GAM) module. This application can extract relevant information for pedestrian target identification tasks and suppress irrelevant information, improving the unidentified problem of occluded and small targets by replacing the GIoU loss function used in the bounding box regression with the α-CIoU loss function. The YOLOv5s-$G^{2}$ network is evaluated on the WiderPerson dataset to ensure its efficacy. Our proposed YOLOv5s-$G^{2}$ network offers a 1.0% increase in detection accuracy and a 13.2% decrease in Floating Point Operations (FLOPs) compared to the existing YOLOv5s network. As a result, the YOLOv5s-$G^{2}$ network is preferable for pedestrian identification as it is both more lightweight and more accurate.

## 1. Introduction

The automated localization of a pedestrian target recognized in an image is known as pedestrian detection. It is a branch of computational vision study and the most significant component of self-driving automobiles, evaluating behavior, human–computer interaction, etc.

In the past, the only way to find people was to manually pull out features through a sliding window and feed them into a classifier. The representative features among the traditional hand-designed features are mainly Haar, Harris [1], HOG [2], Hu moment [3], SIFT [4], and SURF [5]. Furthermore, classification methods are mainly divided into supervised and unsupervised algorithms. Among them, supervised algorithms mainly adopt the Naive Bayes classifier, Support Vector Machine (SVM) [6], or Perception [7]. Then, unsupervised algorithms generally use KMeans [8] and Mean shift [9]. Traditional methods for detecting pedestrians, on the other hand, can not be used in complex scenes because they have to be set up by hand and select regions by sliding a window.

In the early days of deep learning applied to target detection tasks, two-stage detection algorithms based on candidate regions were mainly used. These methods start with a basic analysis of the input image to identify potentially target-bearing regions. Then, after that, these algorithms detected the previously extracted regions by using classification networks, thus completing the target detection task. Typical two-stage detection algorithms are Region-Convolutional Neural Network (R-CNN) series [10,11,12,13,14] and Spatial Pyramid Pooling Network (SPPNet) [15].

One-stage detection algorithms discard the region selection algorithm, use bounding box regression to complete detection and recognition simultaneously, and achieve end-to-end detection and recognition. The You Only Look Once (YOLO) network series [16,17,18,19], the Single Shot MultiBox Detector (SSD) [20] network, and the CenterNet [21] series are examples of this sort of algorithm, which has poorer detection accuracy but quicker detection speed.

Since the introduction of the YOLOv1 network, the YOLO series network has become the mainstream network in target detection. Han et al. [22] improved the detection accuracy of tiny vehicle objects in real time by adding convolutional layers and combining features in the YOLOv2 network. They also handled the gradient explosion issue caused by network depth by applying residual modules. Fu et al. [23] implemented the LeakyReLu function in the YOLOv4 network’s backbone network to redesign it. They achieved network lightweighting by removing the Spatial Pyramid Pooling (SPP) module and network pruning on the backbone network. This model has an accuracy of 1.5% lower than the original model. Zhu et al. [24] changed the number of detection heads of the network from 3 to 4 in order to increase the detection capability of the network for small targets and employed the Transformer encoder in the YOLOv5 network in order to improve the capability of the network to extract features. When compared to the original YOLOv5 network, the FLOPs in this network model rose by 18.3%. These algorithms have made substantial contributions to object identification; however, there is one little issue. Most methods for improving item recognition accuracy will also make the model more complicated and require additional computer resources. Some lightweight network models may efficiently reduce model complexity, but their accuracy suffers as a result.

To address the aforementioned issues, Xu et al. [25] proposed the YOLOv3-promate network model. In order to make the backbone network lighter, they first combined G-Module and Depth-Wish convolution and applied them to the backbone network. They enhanced a network model’s capacity to differentiate between backgrounds and targets by applying attention mechanisms, and they lowered the model’s complexity by deleting certain superfluous channels using network pruning methods. Their network model’s parameters are decreased to a tenth of those in the original YOLOv3 network, and the mAP for vehicle and pedestrian is enhanced by 7%. Yu et al. [26] came up with two new Cross Stage Partial (CSP) modules to replace the CSPDarkNet53 modules in the YOLOv4 network. These new modules use adaptive image scaling algorithms to improve the accuracy of face mask recognition while reducing network complexity. Both of the above methods find a good balance between accuracy and difficulty, but the strategies they use are not just for pedestrian targets. In addition, the YOLOv5 network is more suited for pedestrian detection since it incorporates significant improvements from the YOLOv4 and YOLOv3 networks, which have enhanced real-time performance and detection accuracy.

We propose a YOLOv5s-$G^{2}$ network model with improved accuracy and complexity balance for pedestrian target detection. The main contributions of this paper are as follows:

- To minimize the complexity of the YOLOv5s network without losing precision, we apply Ghost and GhostC3 modules. They can attain a balanced proportion of portability and precision;
- We employ the GAM to network in order to increase the network’s capacity to extract pedestrian target features and construct a more accurate and efficient pedestrian target detector without significantly increasing the network’s complexity;
- We propose to use the α-CIoU loss function in model training. The α-CIoU loss function keeps all the features of the original loss function while emphasizing high IoU targets and generating extra space for optimizing targets at all levels. In addition, utilizing the α-CIoU loss function may increase the correctness of the network model without increasing its computational volume.

The rest of this paper is arranged as follows: Section 2 covers the basic principles of the YOLOv5s network. The structure of the YOLOv5s-$G^{2}$ network described in this study is shown in Section 3, followed by a description of the network’s strategies. Section 4 focuses on the analysis and results of the ablation experiments and comparison experiments of the YOLOv5s-$G^{2}$ network. The work mentioned above is concluded in Section 5.

## 2. YOLOv5s Method

**Figure 1:**
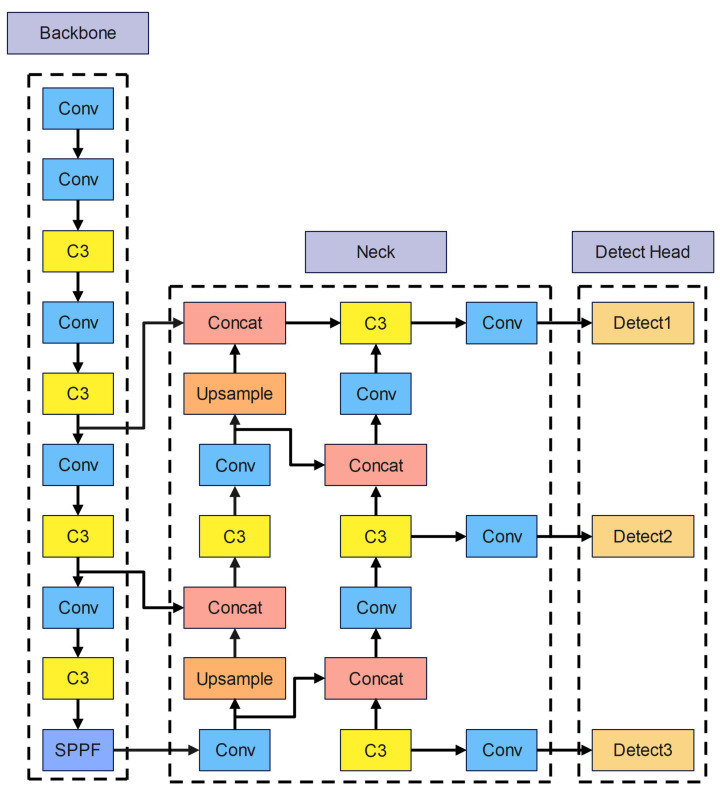
YOLOv5s network architecture.

The width and depth of the residual structure divide the YOLOv5 series networks into five groups: YOLOv5n, YOLOv5s, YOLOv5m, YOLOv5l, and YOLOv5x. All of these five types of networks have the same structure, but the width and depth of the residual structure are different. The data in Table 1 shows the parameters of the five network types and their performance on the MS COCO dataset. Analysis of the data shows that the YOLOv5 network has the better accuracy and complexity balance among the five models. Figure 1 displays YOLOv5s network architecture.

After inputting the original image, YOLOv5s requires the three main methods for processing images mentioned below. First, by merging the four input photos in a random size, crop, and arrangement, mosaic data augmentation improves the identification rate and detection accuracy. In addition, before the original photos are resized to the standard size, black borders that change to fit the size of the photo are added. Third, the Adaptive anchored box calculates the distance between the predicted box and the real box, and then iteratively optimizes the parameters to obtain the appropriate anchor box.

**Figure 2:**
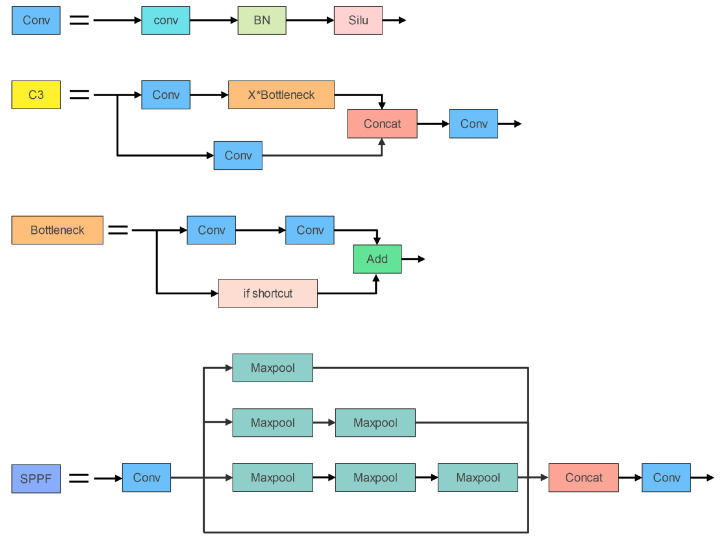
Modules in the YOLOv5s backbone network.

The backbone network’s main modules consist of Conv, C3, and Spatial Pyramid Pooling—Fast (SPPF). Figure 2 depicts the three components’ organizational structure. In addition, the Conv module is the most fundamental component of the YOLOv5s network. The Conv module consists of a convolutional layer, a Batch Normalization (BN) layer [27], and a nonlinear activation function Silu [28]. The C3 module is used for feature extraction in the backbone network. Furthermore, it contains three standard convolutional layers and X bottleneck modules. The Bottleneck module is borrowed from the residual structure of ResNet [29] and is mainly used for feature fusion; The major purposes of the SPPF module are to broaden the perceptual field, extract crucial contextual information, and resolve multi-scale issues.

Path-aggregation Network(PANet) [30] and C3 are applied in the neck for feature fusion. First, PANet employs upsampling to send reliable localization information from the lower layers to the top levels, followed by a bottom-up feature pyramid to convey reliable semantic information from the higher layers. After the feature fusion by PANet, the features passed from neck to head have both robust semantic information and substantial localization information to make the detection more accurate.

Three detection layers are utilized to create three feature vectors of varying sizes. The feature vectors consist of the category possibility of the target object, the object score, and the location of the object’s bounding box for detecting targets.

The loss function comprises three loss functions: classification loss, localization loss, and confidence loss, with the total loss being the weighted sum of the three. The classification loss and localization loss are computed using the binary cross-entropy loss function, whereas the confidence loss is calculated utilizing the G-IoU loss.

## 3. Architecture Design of the YOLOv5s-$G^{2}$ Network

We propose the YOLOv5s-$G^{2}$ network, which is a lightweight pedestrian detection network based on the YOLOv5s network, which can guarantee accuracy with less algorithmic complexity than the original network and effectively reduce the need for computing power.

**Figure 3:**
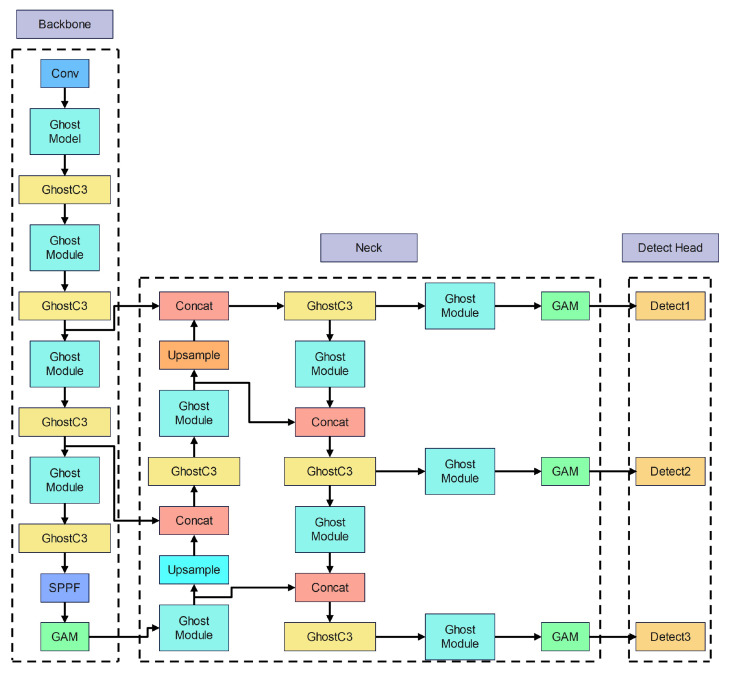
YOLOv5s-$G^{2}$ network architecture.

Figure 3 depicts YOLOv5s-$G^{2}$ network’s architecture. YOLOv5s-$G^{2}$ network applies three different strategies to improve the original YOLOv5s network. First, the C3 module and Conv module in YOLOv5s as shown in Figure 3 were replaced with the more lightweight GhostC3 module and Ghost module. The objective of GhostC3 and Ghost modules is to minimize the model’s complexity even further. Second, the GAM attention module is also utilized in the backbone and neck networks. The GAM attention module enhances the network’s capacity to extract pedestrian features by emphasizing pedestrian-related information, allowing it to recognize pedestrians successfully in various diverse environments. Finally, the network training loss function is modified from the GIou loss function to the α-IoU loss function. During the training procedure, the α-IoU loss function may effectively address the issue of erroneous prediction box localization of pedestrians.

### 3.1. Lightweight Strategy of Network

**Figure 4:**
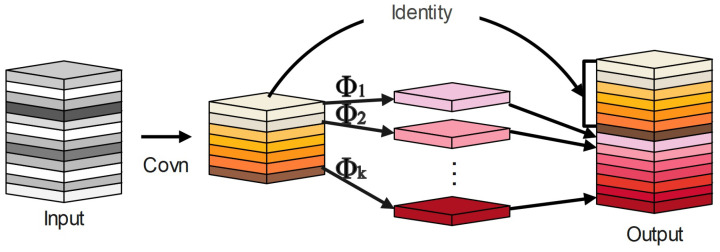
Ghost module.

Han et al. [31] proposed the lightweight module called Ghost Module in 2020. It requires fewer computations and parameters to produce more feature maps. Figure 4 demonstrates how it works.

The Ghost module first applies linear computation to the generated normal convolutional feature map in order to produce a new feature map, and then the two feature maps are combined to obtain a high-dimensional feature map. Thus, it can produce high-dimensional convolution effects while reducing the computational cost of the model.

In the Ghost module, the input features are first convolved by ordinary convolution to generate a fixed number $C^{\prime }$ of intrinsic feature maps $Y\in R^{\omega^{\prime }\times h^{\prime }\times c^{\prime }}$, as shown in Equation (1):

(1) $$Y=X^{*}f$$

$X\in R^{\omega \times h\times c}$ is the input feature, and $f\in R^{k\times k\times \times \times c^{\prime }}$ is the convolution kernel. Moreover, for simplicity, the bias term is ignored. Then, the created $C^{\prime }$ intrinsic features are utilized to calculate the S features associated with them, $y_{ij}$, using a sequence of linear operations, as illustrated in Equation (2).

(2) $$y_{ij}=\phi_{ij}*\left(y_{i}^{\prime }\right),i=1,2,\ldots ,m,j=1,2,\ldots ,s$$

where $y_{i}^{\prime }$ is the *i*-th feature of the intrinsic feature *Y*, and $\phi_{ij}$ is the linear operation to generate the *j*-th associated feature. The feature information $y_{ij}$ generated by the linear operation is connected with the inherent feature *Y* to output the feature information.

**Figure 5:**
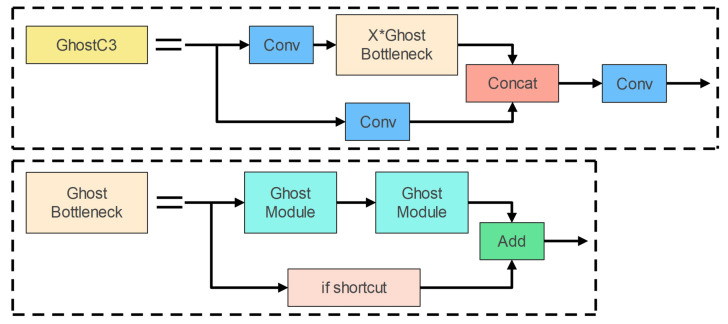
The architecture of the GhostC3 Module.

Ghost bottlenecks are bottleneck structures made out of Ghost modules; they simply employ Ghost modules instead of the bottleneck structure’s standard convolution. Ghost bottlenecks are shown in Figure 5. The Ghost bottleneck layer is analogous to ResNet’s fundamental residual blocks. The first Ghost module is used to expand the number of channels; in order to keep the shortcut consistent, the second Ghost module is used to reduce the number of channels. These two Ghost modules form the Ghost bottleneck layer.

Figure 5 depicts the precise structure of the GhostC3 module, which is created by replacing the bottleneck structure in the C3 module with the Ghost bottlenecks structure with a Stride of 1. Then, We employ the Ghost and GhostC3 modules to substitute the Conv and C3 modules in the network, lowering the model’s complexity.

### 3.2. Global Attention Mechanism

**Figure 6:**
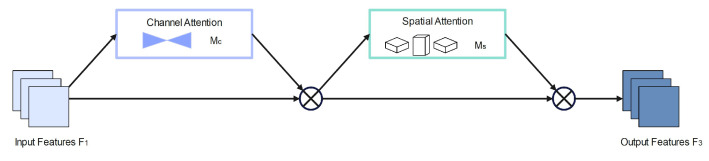
GAM attention module.

The background of the dataset employed in this paper is mostly urban, and the surroundings are intricate and unpredictable. To make the model more accurately represent the characteristics of pedestrians, we employ the GAM module on the ends of the neck and backbone networks. The GAM attention module is a global attention mechanism that enables features to have more global information while decreasing information dispersion in order to enhance neural network performance [32]. Figure 6 depicts the GAM module’s organizational structure.

The GAM global attention module may enhance the network model’s capacity to extract pedestrian features and minimize complicated background interference. The GAM global attention module adopts the channel order in CBAM [33] (convolutional block attention module). First, the CA (Channel Attention) module extracts the channel attention from the input image $F_{1}$. The result is the medium feature map $F_{2}$ with channel focus. The SA (Spatial Attention) module removes the spatial attention from the feature map with channel attention, hence producing the final feature map $F_{3}$. The specific calculation of $F_{3}$ is shown in Equation (3):

(3) $$\begin{array}{cc} \; & F_{2}=M_{c}\left(F_{1}\right)⊗F_{1}\\ \; & F_{3}=M_{s}\left(F_{2}\right)⊗F_{2} \end{array}$$

where $M_{c}$ and $M_{s}$ denote the channel and spatial attention maps, respectively, and ⊗ denotes the multiplication operation performed.

**Figure 7:**
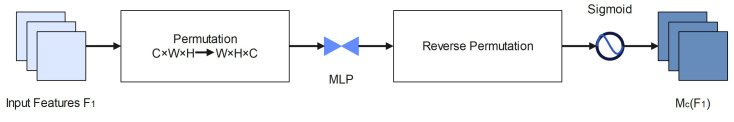
CA Module.

Figure 7 shows the structure of the CA mechanism. The size of the input feature map is C × W × H. The input features’ height and width are denoted by H and W, while the number of channels is denoted by C. The 3D information is kept by using the 3D alignment operation on the input image. After that, a 2-layer MultiLayer Perception (MLP) is applied to the output in order to enhance the cross-dimensional channel–space dependency. Then, the inverse 3D alignment operation is used for the output, and the result will be obtained. The Channel Attention map $M_{c}\left(F_{1}\right)$ is obtained by sigmoid function activation.

**Figure 8:**
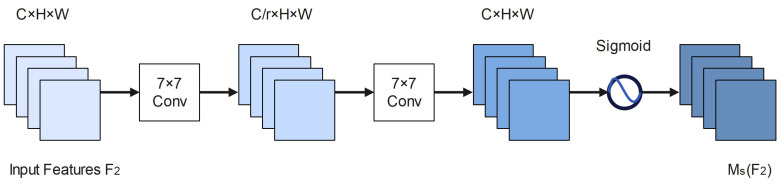
SA module.

The construction of the SA module is shown in Figure 8, with the size of the input medium feature map f2 being C H W. The input features are first spatially fused by two convolutional layers of size 7 × 7 to extract spatial information. We employ group convolution with channel blending wash to avoid the considerable rise in parameters caused by the Spatial Attention module, which may sometimes dramatically increase the number of parameters. The sigmoid function activates the final output to obtain the SA map $M_{s}\left(F_{2}\right)$.

### 3.3. Loss Function Improvement

The GIoU loss employed in the confidence loss has the drawback of having the same G-IoU value if the prediction box is within the target frame. However, its prediction box location is different; therefore, it is hard to locate the ideal prediction box. This is a serious flaw for pedestrian detection. To address this issue, we employ the α-IoU loss function instead of the GIoU loss function to optimize the anchor box [34].

The α-IoU loss introduces a power transformation to the existing IoU loss and proposes a new IoU loss function. α-IoU loss has a Power IoU term and an additional Power canonical term with a single Power parameter α, and α-IoU loss is defined as shown in Equation (4):

(4) $${\mathrm{loss}}_{\alpha -\mathrm{LoU}}=\frac{1-{\mathrm{IoU}}^{\alpha }}{\alpha },\alpha >0$$

In this paper, we mainly use α-IoU loss based on CIoU loss. α-CIoU loss is defined as shown in Equation (5):

(5) $${\mathrm{loss}}_{\alpha -\mathrm{CIOU}}=1-IOU^{\alpha }+\frac{\rho^{2\alpha }\left(b,b^{gI}\right)}{c^{2\alpha }}+{\left(\beta v\right)}^{\alpha }$$

where *c* represents the diagonal distance of the smallest closed area that can contain both the prediction frame and the accurate frame, while ν measures the consistency of the aspect ratio, as defined in Equation (6). β is a positive trade-off parameter with a value as in Equation (7), and $\rho^{2\alpha }\left(b,b^{gI}\right)$ represents the Euclidean distance between the centroids of the prediction frame and the accurate frame:

(6) $$\nu =\frac{4}{\pi }{\left(\arctan\frac{\omega^{gt}}{h^{gt}}-\arctan\frac{\omega }{h}\right)}^{2}$$

(7) $$\beta =\frac{\nu }{(1-IoU)+\nu }$$

$\frac{\omega^{gt}}{h^{gt}}$ and $\frac{\omega }{h}$ represent the respective aspect ratios of the target and predicted frames in Equation (6).

## 4. Results and Discussion

The operating system used in this experiment is Windows 10 Pro for Workstations. The CPU model is Intel Xeon Gold 5218, and the GPU model is Quadro P5000. The deep learning framework is Python 1.10.0, and CUDA version 11.3 is used together with the CUDNN version 8.2.0 deep neural network acceleration library.

### 4.1. WiderPerson Dataset

**Figure 9:**
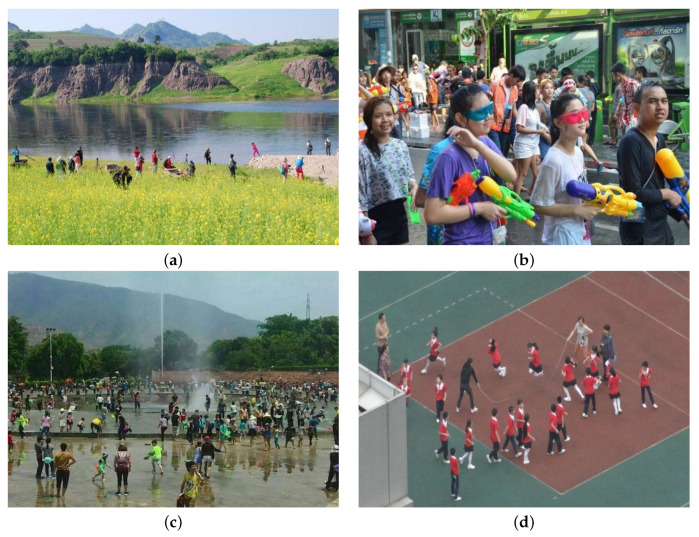
The WiderPerson dataset imgaes: (**a**) Riverside; (**b**) street; (**c**) Square; (**d**) Playground.

This experiment uses the WiderPerson dataset [35], a diverse and dense pedestrian detection dataset with rich foreground and background images and many rich crowd scenes with highly obscured pedestrians. The WiderPerson dataset classifies pedestrians into five categories, the first being pedestrians, which are complete pedestrians. The second category is riders, who ride electric bikes or bicycles. The third category is pedestrians who are partially visible, with all pedestrians being blocked to varying degrees. The fourth category, “ignored region”, consists mainly of objects that look like people but are not people. The fifth category is the crowd, which is densely populated. Since ignored regions and crowds are not people, we remove the labels of these two categories and combine pedestrians, riders, and partially-visible persons into the category of person for the experiment. Since the test data and true frame labels of the original WiderPerson dataset are not disclosed, we utilize 90% of the original training set as our training set, 10% of the original training set as our validation set, and the original validation set as our test set in our experiments. Figure 9 illustrates this data set.

### 4.2. Measurement Indicators

To effectively assess the model’s detection effect, the model’s performance is measured in mAP (mean average precision), while the model’s complexity is stated in FLOPs. The specific expression of mAP is shown in Equation (11):

(8) $$P=\frac{TP}{TP+FP}$$

(9) $$R=\frac{TP}{TP+FN}$$

(10) $$AP=\int_{0}^{1}P\left(R\right)dR$$

(11) $$mAP=\frac{\sum_{i=1}^{k}AP_{i}}{k}$$

In Equation (8), True Positives (*TP*) is the amount of positive samples that the model successfully classified; False Positives (*FP*) is the amount of negative samples that the model wrongly classified as positive; False Negatives (*FN*) is the amount of positive samples missed by the model. In Equation (11), *k* represents the quantity of categories, whereas $AP_{i}$ is the *AP* value of the *i*th category. FLOPs are a measure of how complicated an algorithm or model is and may be used to determine the amount of computation in the model.

### 4.3. Results of the YOLOv5s-$G^{2}$ Network

**Figure 10:**
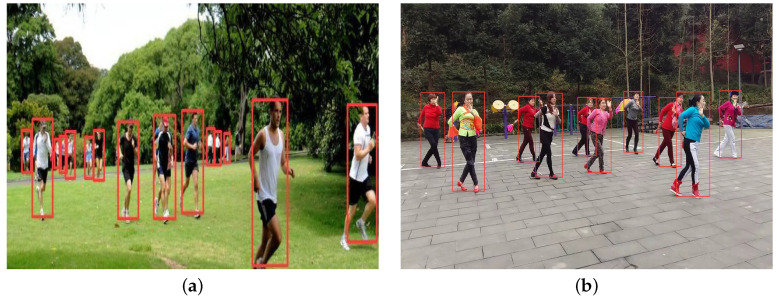
Detection results of the YOLOv5s-$G^{2}$ network: (**a**) Park; (**b**) Square.

The WiderPerson dataset is used to analyze the pedestrian identification results obtained by the YOLOv5s-$G^{2}$ network. Table 2 displays the results of the YOLOv5s-network. YOLOv5s-$G^{2}$ network’s mAP0.5 and mAP0.5:0.95 of pedestrian target and Flops are 76.9%, 48.3%, and 13.7G, respectively. Figure 10 shows the detection performance of YOLOv5s-$G^{2}$ in the in the WiderPerson dataset’s test set.

### 4.4. Ablation Experiments

To evaluate the extent to which different strategies and combinations might enhance the algorithm’s performance, we designed an ablation experiment. All the hyperparameters in the ablation experiment were consistent during all of the model training. The parameter design of the ablation experiment is shown in Table 3.

The results of the ablation experiments on the YOLOv5s-$G^{2}$ network on the widerperson dataset are shown in Table 4. The experimental ablation data showed that the addition of each strategy produced different degrees of optimization of the final structure. Experiment 2 shows that the network reduces FLOPs to 52% after the Ghost module is introduced, but mAP0.5 and mAP0.5:0.95 decrease only 1.4% and 1.6%, which demonstrates that the Ghost module may successfully decrease the algorithm’s complexity with the sacrifice of some accuracy. In Experiment 3, although FLOPs increased by 35.4% after the GAM attention mechanism module was applied, mAP0.5 of the network increased by 0.7%. The results from Experiment 3 indicate that the implementation of the GAM attention mechanism may strengthen the backbone network’s capacity to extract features and pay attention to a large amount of swiftly submerged semantic information, thus improving the network’s accuracy. In Experiment 4, α-CIoU loss improves the mAP0.5 and mAP0.5:0.95 of the network by 1.7% without increasing the network complexity. α-CIoU loss decreases the regression loss of the prediction box and increases the regression accuracy, which may be quite beneficial for enhancing the network’s performance for pedestrian identification.

The different strategy combinations also essentially showed positive optimization on the overall network performance. By comparing the data of Experiment 5, Experiment 2, and Experiment 3, it can be seen that the mAP0.5 and mAP0.5:0.95 of the network with GAM module and α-CIoU loss improved by 1.7% and 2.1% compared to the network with GAM module and improved by 0.7% and 0.3% compared to the network with α-CIoU loss. The comparison of Experiment 6 and Experiment 7 with Experiment 2 shows that both the GAM attention mechanism and α-CIoU loss can be applied to the network after introducing the Ghost module. Compared with the network introduced as Ghost module only, the mAP0.5 and mAP0.5:0.95 of the network improved by 1.1% and 0.8%, respectively, after adding GAM attention, and the mAP0.5 and mAP0.5:0.95 of the network improved by 2.0% and 2.1%, respectively, after adding α-CIoU loss.

Finally, the final network YOLOv5s-$G^{2}$ with all three strategies simultaneously improves by 1.0% and 1.2% compared to the original YOLOv5s network with mAP0.5 and mAP0.5:0.95, respectively. Moreover, the FLOPs are reduced by 13.3%, which shows that the simultaneous adoption of the three strategies may somewhat weaken the optimization of individual strategies, but at the same time, maintain the better values of accuracy and complexity, achieving a more desirable balance in the setting of this paper.

### 4.5. Comparative Experiment

We chose the YOLOV3-tiny network, YOLOV4-tiny network, and YOLOX-tiny3 network for comparative testing to ensure that our proposed YOLOv5s-$G^{2}$ network produces superior results. The data set and settings utilized for the comparative experiments are consistent with the ablation experiments, as are the parameters used for the four networks. Table 5 displays the findings of the comparative trials.

The YOLOv3-tiny network uses two main lightweighting strategies. First, the YOLOv3-tiny network removes the residual structure in the backbone of the YOLOv3 network. Second, the YOLOv3-tiny network is deleting one detection head in the YOLOv3 network and keeping only two detection heads. However, since YOLOv3-tiny network’s lightweighting strategy is only to make the network lighter, it sacrifices a certain amount of detection accuracy. The mAP0.5 and mAP0.5:0.95 of the YOLOv3-tiny network, on the other hand, are 10.2% and 10.7% lower, respectively, compared to the YOLOv5s-$G^{2}$ network. The experimental results demonstrate that the YOLOv5s-$G^{2}$ network has more balanced complexity and detection accuracy than the YOLOv3-tiny network. The lightweighting strategies employed in this study for the YOLOv5s-$G^{2}$ network are not only a reduction of the network, which explains why. The YOLOv5s-$G^{2}$ network retains the basic architecture of the original YOLOv5s network as much as possible, and the network is made lighter by replacing the Conv module in the YOLOv5s network with the lighter Ghost module. In addition, the YOLOv5s-$G^{2}$ network applies the attention mechanism and α-CIoU loss to alleviate the problem of accuracy degradation caused by being lightweight.

The YOLOv4-tiny network uses a similar lightweighting strategy as the YOLOv3-tiny network, by making specific deletions to the original network. First, the YOLOv4-tiny network changes the backbone network activation function to a LeakyReLu function and also deletes a certain amount of residual structure. However, unlike the YOLOv3-tiny network, the YOLOv4-tiny network retains three residual structures. Secondly, like the YOLOv3-tiny network, only two detection heads and one feature fusion pyramid are retained. Since the YOLOv4-tiny network uses a lightweighting strategy like YOLOv3-tiny, the YOLOv4-tiny network suffers from the same accuracy degradation problem. According to Experiment 2 and Experiment 4, the YOLOv4-tiny network has 10.5% and 16.8% less mAP0.5 and mAP0.5:0.95 than the YOLOv5s-$G^{2}$ network in addition to having 2.4 G more FLOPs than the YOLOv5s-$G^{2}$ network. Thus, it can be seen that the lightweighting strategy used by YOLOv4-tiny is unsuitable for pedestrian detection.

The lightweighting strategy used in the YOLOX-tiny network maintains the original YOLOX network framework but decreased channels in the network, thus making the network lighter. Comparing the data from Experiment 3 and Experiment 4 shows that the YOLOX-tiny network has only 1.4% and 4.5% lower mAP0.5 and mAP0.5:0.95 compared to the YOLOv5s-$G^{2}$ network. The FLOPs of the YOLOX-tiny network are also 1.4 G higher than those of the YOLOv5s-$G^{2}$ network. These data show that the YOLOv5s-$G^{2}$ network uses a better strategy for pedestrian detection than the YOLOX-tiny network.

## 5. Conclusions

The YOLOv5s-$G^{2}$ network is a lightweight pedestrian detection network proposed in this paper. In the YOLOv5s-$G^{2}$ network, we have the lightweight GhostC3 and Ghost modules, which may minimize network complexity while maintaining network detection accuracy. We apply the GAM to YOLOv5s-$G^{2}$ network. The attention mechanism GAM effectively enhances the capability of YOLOv5s-$G^{2}$ to extract pedestrian feature information. The α-CIoU loss function is proposed to replace the GIoU loss function. α-CIoU loss can improve the regression accuracy by reducing the regression loss of the loss function. Therefore, the α-CIoU loss could significantly address the missing detection of tiny objects and localization issues for the Prediction Box in complicated backgrounds. Ablation experiments on the WiderPerson dataset show that the combination of strategies in the YOLOv5s-$G^{2}$ network can enhance pedestrian recognition accuracy while minimizing network complexity compared to the original YOLOv5s. It is also demonstrated that the YOLOv5s-$G^{2}$ network achieves a better balance of accuracy and complexity than other lightweight networks.

In the future, we will further reduce the complexity of the network by removing redundant convolutional layers using pruned networks to ensure that the YOLOv5s-$G^{2}$ network will be capable of substantially decreasing the computational power requirements of the platform and be more easily deployed on resource-limited devices.

## Figures and Tables

**Table 1:** Comparison of YOLOv5 series networks’ performance.

Method	Image Size	mAP0.5(%)	mAP0.5:0.95(%)	FLOPs(G)
YOLOv5n	640 × 640	45.7	28.0	4.5
YOLOv5s	640 × 640	56.8	37.4	16.5
YOLOv5m	640 × 640	64.1	45.4	49.0
YOLOv5l	640 × 640	67.3	49.0	109.1
YOLOv5x	640 × 640	68.9	50.7	205.7

**Table 2:** Results of the YOLOv5s-$G^{2}$ network.

Method	mAP0.5(%)	mAP0.5:0.95(%)	FLOPs(G)
YOLOv5s-$G^{2}$	76.9	48.3	13.7

**Table 3:** Ablation experiment parameters.

Types	Value
Epoch	300
Batch Size	16
Input Image Size	640 × 640
Optimizer	SDGM
Initial Learning Rate	0.01

**Table 4:** Results of ablation experiments. In particular, Experiment 1 shows the results of the Standard YOLOv5s network model.

No.	Ghost Module	GAM	α-CIoU	mAP0.5(%)	mAP0.5:0.95(%)	FLOPs(G)
1				75.9	47.1	15.8
2	△			74.5	45.5	8.2
3		△		76.6	47	21.4
4			△	77.6	48.8	15.8
5		△	△	78.3	49.1	21.5
6	△	△		75.6	46.3	13.7
7	△		△	76.5	47.6	8.1
8	△	△	△	76.9	48.3	13.7

**Table 5:** Results of the comparison experiment.

Method	mAP0.5(%)	mAP0.5:0.95(%)	FLOPs(G)
YOLOv3-tiny	66.7	37.6	12.9
YOLOv4-tiny	66.4	31.5	16.1
YOLOX-tiny	75.1	43.8	15.1
YOLOv5s-$G^{2}$	76.9	48.3	13.7

## Data Availability

Publicly available datasets were analyzed in this study. This data can be found here: http://www.cbsr.ia.ac.cn/users/sfzhang/WiderPerson/ (accessed on 22 July 2022).
